# Epigenetic Silencing of Apoptosis-Inducing Gene Expression Can Be Efficiently Overcome by Combined SAHA and TRAIL Treatment in Uterine Sarcoma Cells

**DOI:** 10.1371/journal.pone.0091558

**Published:** 2014-03-11

**Authors:** Leopold F. Fröhlich, Maria Mrakovcic, Claudia Smole, Pooja Lahiri, Kurt Zatloukal

**Affiliations:** 1 Molecular Pathology Laboratory, Medical University of Graz, Graz, Austria; 2 Center for Medical Research, Medical University of Graz, Graz, Austria; University of Quebec at Trois-Rivieres, Canada

## Abstract

The lack of knowledge about molecular pathology of uterine sarcomas with a representation of 3–7% of all malignant uterine tumors prevents the establishment of effective therapy protocols. Here, we explored advanced therapeutic options to the previously discovered antitumorigenic effects of the histone deacetylase (HDAC) inhibitor suberoylanilide hydroxamic acid (SAHA) by combined treatment with the tumor necrosis factor-related apoptosis-inducing ligand (TRAIL/Apo-2L). In addition, we investigated the uterine sarcoma cell lines, MES-SA and ESS-1, regarding the underlying molecular mechanisms of SAHA and TRAIL-induced apoptosis and their resistance towards TRAIL. Compared to single SAHA or TRAIL treatment, the combination of SAHA with TRAIL led to complete cell death of both tumor cell lines after 24 to 48 hours. In contrast to single SAHA treatment, apoptosis occured faster and was more pronounced in ESS-1 cells than in MES-SA cells. Induction of SAHA- and TRAIL-induced apoptosis was accompanied by upregulation of the intrinsic apoptotic pathway via reduction of mitochondrial membrane potential, caspase-3, -6, and -7 activation, and PARP cleavage, but was also found to be partially caspase-independent. Apoptosis resistance was caused by reduced expression of caspase-8 and DR 4/TRAIL-R1 in ESS-1 and MES-SA cells, respectively, due to epigenetic silencing by DNA hypermethylation of gene promoter sequences. Treatment with the demethylating agent 5-Aza-2'-deoxycytidine or gene transfer therefore restored gene expression and increased the sensitivity of both cell lines against TRAIL-induced apoptosis. Our data provide evidence that deregulation of epigenetic silencing by histone acetylation and DNA hypermethylation might play a fundamental role in the origin of uterine sarcomas. Therefore, tumor growth might be efficiently overcome by a cytotoxic combinatorial treatment of HDAC inhibitors with TRAIL.

## Introduction

Uterine sarcomas consist of several distinct histiological subtypes and are rare entities as they comprise only 3–7% of all uterine cancers but account for 20% of deaths [1]. The most common types of the mesenchymal subgroup, classified according to the World Health Organization in 2003, include carcinocarcinomas (∼ 40% of cases), leiomyosarcomas (∼ 40% of cases), endometrial stromal sarcomas (ESS; 10–15% of cases) and undifferentiated sarcomas (5–10% of cases) [2], [3]. Patients with unresectable advanced uterine sarcomas have a very poor prognosis because no effective chemotherapeutic protocols exist [4]. One reason for this might originate in the lack of information regarding molecular pathogenetic mechanisms of these tumors. Due to the rareness of the disease only few tumors have so far been characterized at the molecular level. Furthermore, there are currently hardly any established primary human uterine sarcoma cell lines available, in particular for ESS that can be used to investigate disease mechanisms and potential therapies.

Epigenetic silencing of gene expression is an important oncogenic mechanism [5]. Causative mechanisms involve both, loss and gain-of-methylation of DNA [6], as well as changed patterns of histone modifications [7]. By alteration of DNA methylation, in particular hypermethylation of critically important genetic regulatory elements, e.g. CpG islands located in the promoter regions of genes, the cancer cell achieves deregulation of gene expression [8]. A second way of epigenetic gene silencing, is provoked by the upregulation of HDAC expression which has a critical role in mediating a transcriptionally inactive chromatin structure [9]. As a heterogeneous group of enzymes, HDACs act mainly as gene expression regulators, by deacetylating the lysine residues in the amino-terminal tails of histone proteins [7]. Some sarcomas are associated with chromosomal translocations for which antitumor activity by HDAC inhibitors has been demonstrated. This can occur through abnormal recruitment of HDACs to gene promoters [10]. Although still very unclear, one mechanism that emerges here are histone modifications (eg. acetylation or methylation) in combination with recruitment of polycomb-group complex repressor proteins (PcGs) initiated by fusion oncoproteins. Several translocation events and resultant gene fusions involving PcGs with the most common variant joining parts of the JAZF1 gene to the PcGJJAZ1/SUZ12 were also detected in ESS [11].

Previously, the consistent upregulation of expression of the class II enzyme HDAC2 (80% in comparison to non-neoplastic endometrial stroma) was demonstrated in ESS by our group [12]. Furthermore, the HDAC inhibitor SAHA (marketed as Vorinostat or Zolinza) significantly prevented tumor cell proliferation by increasing expression of the cell cycle kinase p21^WAF1^ and decreasing expression of HDAC2 and 7 in ESS-1 cells [13]. Upon 48 hours of SAHA treatment, both studied cell lines, MES-SA [14], which was derived from the sarcomatous element of a mixed müllerian tumor (carcinocarcinoma), as well as ESS-1 [15], which was isolated from low grade ESS, lost their colony forming capability. In contrast, exposure of non-tumorigenous human endometrial stromal cells (HESCs) to SAHA resulted only in slightly decreased cell proliferation. Moreover, xenografted tumors of MES-SA showed a more than 50% growth reduction upon SAHA treatment in comparison to a control group by pronounced activation of apoptosis [13]. ESS-1 cells, however, underwent predominantly autophagy-mediated cell death upon SAHA treatment with decreased expression of the autophagic molecular determinants mTOR and phospho-S6 ribosomal protein (S6rp) [16].

The HDAC inhibitor SAHA can inhibit cell proliferation by blocking progression through the G1 or G2/M phases of the cell cycle, suppress angiogenesis, and induce cellular differentiation, apoptosis, as well as autophagy in cancer cells as a single agent [16], [17]. Its inhibitory activity enhances the acetylation of histones which induces chromatin relaxation, and results in altered expression of about 2–5% of expressed genes in various tumor cell lines. SAHA is an approved drug for cutaneous T-cell lymphoma [18] which is currently tested alone or in combination with other medical substances. The variety of clinical trials include patients with other solid tumors, such as mesothelioma, medulloblastoma, prostate and thyroid cancer [19].

One of the drugs used in combination with SAHA is TRAIL, a member of the tumor necrosis factor (TNF) superfamily of death inducing ligands. TRAIL induces apoptosis by selective binding to two membrane-bound apoptosis-inducing receptors, TRAIL receptor-1 (DR4/TRAIL-R1) and TRAIL receptor-2 (DR5/TRAIL-R2). Activation of one of the death receptors results in trimerization of the receptor and formation of the death-inducing signalling complex (DISC) [20]. Once the initiator caspase-8 is activated at the DISC, it can directly activate pro-caspase-3. This engagement of caspase-3, via the socalled extrinsic pathway, leads in turn to the ultimate initiation of apoptosis and the cleavage of further downstream substrates e.g. PARP-1 (poly ADP-ribose polymerase-1). A second possibility of transmitting the apoptotic signal emanating from DISC-activated caspase-8 is via the intrinsic (mitochondrial) pathway. The truncated form of BID (tBID) translocates to mitochondria where it reduces the mitochondrial membrane potential causing cytochrome-C release in turn [21]. A complex with apoptotic protease activating factor 1 (APAF-1) and extramitochondrial cytochrome-C, which is called the apoptosome, furtheron, leads to activation of pro-caspase-9. Proteolytic cleavage of pro-caspase-3 by caspase-9 again leads to the full-fledged commitment to induce programmed cell death and presumably amplifies caspase-8 and -9 initiation signals [22].

As a naturally occurring endogenous defense mechanism of the cell in the elimination of cancer cells, TRAIL was found to be an effective and potent drug in cancer therapy [23]. The advantage of the cytokine TRAIL is that it is predominantly targeting cancer cells, while normal cells are left unharmed. Currently, both, recombinant TRAIL proteins and TRAIL receptor agonistic antibodies are under evaluation in a number of clinical trials in single or combined treatments, showing encouraging antitumor activities and mild side effects. Unfortunately, resistance to TRAIL therapy is frequently encountered requiring combined treatments with sensitizing agents. Therefore, in recent years, HDAC inhibitors that reverse aberrant epigenetic changes have emerged as a potential strategy to sensitize cancer cells for TRAIL-induced apoptosis. SAHA and TRAIL were demonstrated to possess augmented apoptosis-inducing potential in combination, in an increasing list of human cancer cell lines, such as leukemia [24], prostate cancer [25], breast tumor [26], melanoma [27], Ewing sarcoma [28], and hepatocellular carcinoma [29]. Up to date, synergistic tumor cell death has been reported in a variety of human cancers using different HDAC inhibitors in combination with TRAIL [30].

Here, we further focussed on the establishment of an improved therapeutic concept for the previously studied uterine sarcomas cell lines and investigated the cause of their cell death resistance. By employing combinatorial effects of SAHA and TRAIL, we obtained insights into the molecular events that lead to the synergistic interaction of the HDAC inhibitor and death receptor ligand. Our *in vitro* data suggest that the combination of Vorinostat with TRAIL or a DNA demethylating agent (decitabine) might provide the basis for an effective therapy for the treatment of ESS or uterine sarcomas in general.

## Materials and Methods

### Chemicals

SAHA was purchased from Alexis Biochemicals (Lausen, Switzerland). A 10 mM stock solution was prepared with dimethyl sulfoxide (DMSO) and stored at -20°C. DMSO never exceeded a concentration of 0.006% in any experiment and therefore did not interfere with cell growth. RhTRAIL/APO-2L was purchased from eBiosciences (Vienna, Austria; 10 ng/μl) or Biomol (Hamburg, Germany; 1 μg/μl). Inhibitors for caspase-3 and -7 (Z-DEVD-FMK), -8 (Z-IETD-FMK), -9 (Z-LEHD-FMK) and the caspase-family inhibitor (Z-VAD-FMK) were obtained from BioVision (CA, USA). 5-Aza-2'-deoxycytidine (5-Aza-dC) was received from Sigma-Aldrich (Vienna, Austria). Restriction enzymes and DNA markers (Gene ruler 50 bp DNA ladder, λ*Bst*91I marker) were purchased from Fermentas (St. Leon-Rot, Germany).

### Cell culture

The human uterine sarcoma cell line MES-SA, established by Harker *et al.* [14], was obtained from ATCC (ATCC Nr. CRL-1976) and cultivated in McCoys 5a medium (Biochrom AG, Berlin, Germany). The human ESS cell line, ESS-1 [15], was purchased from the German Collection of Microorganisms and Cell Cultures (Braunschweig, Germany) and cultivated in RPMI 1640 medium (PAA, Pasching, Austria). Human, non-neoplastic endometrial stromal cells (HESC; ATCC No. CRL-4003) were established by Krikun *et al.* [31] and grown in a 1∶1 v/v mix of DMEM (LifeTech, Vienna, Austria) and Ham´s F12 (PAA, Pasching, Austria). All cell culture media were additionally supplemented with heat-inactivated fetal calf serum (10%, v/v), 100 units/ml penicillin, and 100 μg/ml streptomycin. Cells were cultured under standard conditions (37°C, 5% CO_2_, and 95% humidity). Experiments were only conducted with cell passage numbers below 20.

### MTS assay

Cell growth and viability were determined by MTS assay using the CellTiter 96 AQueous Non-Radioactive Cell Proliferation Assay (Promega GmbH; Mannheim, Germany). The assay measures mitochondrial dehydrogenase activity in living cells by bioreducing a tetrazolium compound [3-(4, 5-dimethylthiazol-2-yl)-5-(3-carboxymethoxyphenyl)-2-(4-sulfophenyl)-2H-tetrazolium, inner salt; MTS] into a formazan product. The quantity of formazan that is directly proportional to the number of living cells in culture was directly measured from 96-well assay plates without additional processing. For the individual assays, 5×10^3^ cells per well were seeded in 96-well plates, incubated at 5% CO_2_ and 37°C, and the appropriate treatment was started 24 hours later. Each experiment included interference controls for each treatment in the maximal concentration applied as well as untreated and medium controls. After 12 to 48 hours of treatment, 20 μl of MTS solution was added to each well. Plates were then incubated for 2 more hours at 37°C, whereafter the absorbance of the formazan product was recorded at 490 nm using a SpectraMax Plus 384 microplate reader (Molecular Devices, CA, US). Results are expressed as percentage of relative viable cells as compared to untreated control cells. In case caspase inhibitors were administered, they were added directly to the cells 1 hour before beginning treatment, at a concentration of 10 μM.

### LDH assay

Release of lactate dehydrogenase (LDH) was measured using the CytoTox-ONE Homogeneous Membrane Integrity Assay (Promega GmbH; Mannheim, Germany) according to the manufactureŕs instructions. In brief, 25 μl of the cell culture supernatant was transferred into a 96 well plate and mixed with an equal volume of the provided substrate mix. After 10 minutes incubation at room temperature (RT), the reaction was stopped by adding 0.2 volumes of the provided stopping reagent. The reaction was evaluated on a FLUOstar Optima fluorescence photometer with an excitation/emission wavelength of 560/590 nm. For a positive control, cells were treated with a lysis solution of equal amounts of Triton X-100 and 70% ethanol for 10 min at RT. Results are expressed as percentage of relative cell numbers of the lysis control.

### Caspase-3 and -7-activation

Caspase activity in the supernatant was determined by using the Caspase-Glo 3/7 Assay (Promega; Mannheim, Germany). An equal volume of assay reagent was added to the medium and the plate was then incubated at RT for 30 minutes protected from light. After incubation, the whole supernatant was transferred to a 96 well plate suitable for luminescent detection and was measured on a LUMIstar Optima luminescence photometer (BMG Labtech; Offenburg, Germany). For the individual assays, 5×10^3^ cells per well were seeded in 96-well plates, incubated at 5% CO_2_ and 37°C, and the appropriate treatment was started 24 hours later. Each experiment included interference controls for each treatment at the maximal concentration applied as well as untreated and medium controls. In case caspase inhibitors were administered to the cells, they were added directly to the cells 1 hour prior to the start of the treatment at a concentration of 10 μM. Results are expressed as percentage of caspase 3/7 activity in samples related to those of untreated control cells.

### Mitochondrial transmembrane potential (Δψ_m_)

Δψ_m_ was determined in uterine sarcoma cells by the JC-1 Mitochondrial Membrane Potential Kit (Biotium; Hayward, CA, USA). Cells were cultured and stained with 5, 5′, 6, 6′-tetrachloro-1, 1′, 3, 3′-tetraethylbenzimidazolyl-carbocyanine iodide (JC-1). Upon collapse of the Δψ_m_, JC-1 molecules can enter mitochondria where they form red J-aggregates if they exceed a critical concentration. JC-1 dye that cannot enter mitochondria remains in the cytoplasm in a green fluorescent monomeric form. Accumulation of the JC-1 dye in mitochondria is therefore potential-dependent, which can be measured by the ratio of fluorescence emission shift from green (∼529 nm; low Δψ_m_) to red (∼590 nm; high Δψ_m_). Thus, mitochondrial depolarization in dead cells, or cells undergoing apoptosis, is indicated by a decrease in the red/green fluorescence intensity ratio. 1×10^4^ cells per well were seeded in 96-well plates, incubated at 5% CO_2_ and 37°C, and treatment was started after 24 hours for 4, 8, and 24 hours. Before the JC-1 staining protocol was applied, cells were washed twice in 1 x phosphate buffered saline (PBS) to get rid of dead cells. Fluorescence was finally monitored in a FLUOstar Optima fluorescence photometer with an excitation/emission wavelength of 550 nm/600 nm for red fluorescence and 485 nm/535 nm for green fluorescence. The ratio of the red/green fluorescence was calculated and presented in arbitrary units.

### Cytofluorometric analysis

Quantification of early apoptotis was performed by fluorescence activated cell sorting analysis (FACS) using the Alexa Fluor 488 Annexin V (AnnV)/Dead Cell Apoptosis Kit (Invitrogen/LifeTechologies, Vienna, Austria). Simultaneous staining with AnnV-FITC and the live/dead dye propidium iodide (PI) in the provided binding buffer is typically used to allow the discrimination of intact cells (PI negative, AnnV-FITC negative), early apoptotic cells (PI negative, AnnV-FITC positive), and late apoptotic cells (PI positive, AnnV-FITC positive), as well as dead (necrotic) cells (PI positive, AnnV-FITC negative). 1×10^5^ cells were plated out on a 6-well plate and treatment was started after 24 hours. After further incubation for 24 hours at 5% CO_2_ and 37°C, attached, as well as floating dead cells were harvested, combined, and washed in cold 1× PBS, before the protocol was continued. The fluorescence emission of 1×10^4^ stained cells were finally measured by a flow cytometer (BD FACSCalibur™, BD Biosciences) at 530 nm and 575 nm using 488 nm excitation. SAHA treated cells were included as a positive control and untreated cells as negative control. Representative measurements, where similar results were obtained in three independent experiments, are shown.

### YoPro-1/PI staining

For visualizing apoptotic cells, the Vybrant Apoptosis Assay Kit 4 (Molecular Probes/LifeTechnologies, Vienna, Austria) was applied according to the instruction manual. 150×10^3^ cells were plated out on 6-well borosilicate glass plates (Asahi Glass Co., Tokyo, Japan) and treatment was started after 24 hours followed by 8 hours of incubation at 5% CO_2_ and 37°C. Then, the cells were washed once in 1 x PBS and incubated for 10 minutes on ice with 400 μl assay buffer (containing 1 μl YO-PRO R-1 stock solution and 1 μl PI stock solution per 1 ml of cell suspension) protected from light. Cells treated with 500 μM H_2_O_2_ presented the positive control while untreated cells were included as a negative control. Finally, cells were viewed at the confocal laser scanning microscope and analysed with LSM510 Meta (Zeiss, Oberkochen, Germany). Images were acquired at an excitation wavelength of 488 nm (using a BP 505–530 nm band-pass detection filter for the green channel) and of 543 nm (in conjunction with LP560) for the red channel (PI). After staining, early (primary) apoptotic cells of the cell population show green fluorescence, late (secondary) apoptotic cells show green and red fluorescence, dead or necrotic cells show only red fluorescence, and live cells show little or no fluorescence. Cells positive for both dyes representing apoptotic and dead or necrotic cells can be seen in yellow/orange color in merged confocal microscopy. All observations were reproduced at least three times in independent experiments.

### Western blot analysis

Attached and floating dead cells were collected (by cell scraper), washed with ice-cold 1 x PBS, shortly sonified and homogenized by a MagNA Lyser instrument (Roche-Diagnostics, Penzberg, Germany) at 6500 rpm for 30 seconds. Then cell lysates were prepared and the protein concentration determined as previously described [32]. Protein lysates were separated by SDS-PAGE (NuPage 4–12% or 12% Bis-Tris gels; Novex/Life Technologies, Vienna, Austria) and transferred to nitrocellulose membrane (Bio-Rad, Vienna, Austria). Following antibodies and concentrations/dilutions were used: rabbit anti DR4 (1 μg/ml)/1:375; rabbit anti DR5 (1 μg/ml)/1:375 (Abcam, Cambridge, UK); rabbit anti Cleaved Caspase-3, -6, -7, -8/1∶1000; and rabbit anti Cleaved poly-ADP-ribose polymerase-1 (PARP-1)/1∶1000 (Cell Signaling, Frankfurt, Germany). As secondary antibodies, we used rabbit anti-mouse and swine anti-rabbit HRP-coupled antibodies at a final concentration of 1 μg/ml (DAKO, Copenhagen, Denmark). An overnight incubation with gentle shaking at 4°C was used for all primary antibodies, followed by washing and 2 hours incubation at RT with secondary antibodies. Specific protein bands were visualized by enhanced chemiluminescence assay (ECL; Amersham Biosciences, Buckinghamshire, UK). All Western blots were probed with a polyclonal rabbit anti β-tubulin antibody (#2146/1∶1000) (Cell Signaling) or mouse anti β-tubulin antibody (ab21058/1∶1000) (Abcam, Cambridge, UK) to demonstrate equal loading of protein samples.

### Reverse transcription and qRT-PCR analysis

RNA was isolated with the Trizol RNA Isolation Reagent (Life Techologies/Invitrogen, Vienna, Austria) according to the instructions and dissolved in Aqua bidest. A total of 2 μg of RNA were transcribed using the High Capacity Reverse Transcription Kit (Applied Biosystems, Foster City, CA, US). Power SYBR Green PCR Master Mix from Applied Biosystems was used to perform quantitative real-time polymerase chain reaction (qRT-PCR) analysis on an AB PRISM 7900HT cycler using previously described primers for DR4 [33] and for caspase-8 [34]. qRT-PCR for DR4, DR5, Dc-R1, Dc-R2, and caspase-8 was performed with primer sequences and PCR conditions as previously described [35]. Amplification of beta-actin (Stratagene, Heidelberg, Germany) was used as standard for cDNA integrity and equal gel loading. Specificity of PCR-reaction products was controlled by DNA sequencing. PCR products were run on a 1.5% agarose gel, stained with ethidium bromide, and visualized by UV illumination.

### Methylation-specific PCR and bisulfite sequencing

Genomic DNA was isolated according to standard methods using a proteinase K digest. For bisulfite sequencing analysis 1 μg of purified genomic DNA was treated with the EpiMark Bisulfite Conversion kit (NEB, Frankfurt, Germany) which, after bisulfite conversion and the subsequent desulphonation reaction, converts cytosine residues to uracil, but leaves 5-methylcytosine residues unaffected. Methylation-specific PCR (MSP) [36] and bisulfite sequencing [37] were performed as previously described [38]. Published primers corresponding to CpG-rich regions of the 5' flanking region of human *caspase-8* (nucleotides – 300 to – 697) [8], [39] and *TNSFR10A* (DR4) genes (nucleotides – 27 to – 267) [33] were used to amplify PCR products. MSP amplicons were run on a 2% agarose gel, stained with ethidium bromide, and visualized by UV illumination. Amplicons for bisulfite sequencing were tested for full conversion and methylation status by combined bisulfite restriction analysis before they were subcloned in a pCR4-TOPO vector and sequenced. Genomic DNA, not treated with bisulfite (unmodified), could not be amplified with either the methylated or the unmethylated primers, and was used as a negative control.

### DR4 and caspase-8 gene transfer

Expression vectors containing human full-length cDNAs for caspase-8 (IMAGE ID 5217189; IRATp970F0250D) or TNFRSF10A (Image ID 3857315; IRATp970H0615D) were obtained from Source BioScience LifeSciences (Nottingham, UK). The cDNAs were obtained as purified plasmid DNA and inserted in the pCMV-Sport6 vector. Either cDNA containing expression vectors or vector without insert (mock control) were transfected using Lipofectamine 2000 transfection reagent (Invitrogen/Life Technologies). Cotransfection with pEGFP-N1 plasmid (Clontech, Mountain View, CA, USA; GenBank accession no. U55762) was performed for transient transfection to identify transfected cells (>85% transfection efficiency). The appropriate treatment was started 24 hours after transfection.

### Additive model

The effect of combinations of drugs used in this study was assessed using an additive model [40]. The effect on cell viability by each treatment alone is multiplied together to give the expected value (E) for the combination of drugs. The actual effect on cell viability when drugs are combined is the observed value (O). The additive model predicts whether the combined effects of two or more drugs are synergistic when the ratio O/E < 0.8, additive when O/E  =  0.8–1.2, or subadditive when O/E > 1.2.

### Statistical analysis

If not stated otherwise, all values represent means of at least three independent experiments ± standard deviation (SD) of at least triplicate measurements. Statistical significance (*p*) was calculated using paired Student's *t* test. A *p-*value ≤0.05 was considered statistically significant.

## Results

### Combined SAHA and TRAIL treatment potentiates the cytotoxic effects in uterine sarcoma cells

In previous studies, our group verified that the HDAC inhibitor SAHA exerts cytotoxicity on uterine sarcoma cells. The number of cells were decreased in a time- and dose-dependent manner whereby an established working dose of 3 μM SAHA significantly reduced ESS-1 cells by 80% [16] and MES-SA cells by 48% [13] after 24 hours of treatment, and inhibited the G1/S transition *in vitro*. During further experiments with combined SAHA and TNF-alpha (TNF-α) treatment, we noticed a rapid cytotoxic effect on our investigated tumor cells (data not shown). As TRAIL acts in a similar way as TNF-α by binding to the cell membrane receptors DR4/TRAIL-R1 and/or DR5/TRAIL-R2 and by transmitting an apoptotic signal via their cytoplasmic death domain [20], we investigated whether a SAHA/TRAIL combination treatment enhances cell death in uterine sarcoma cell lines.

**Figure 1:**
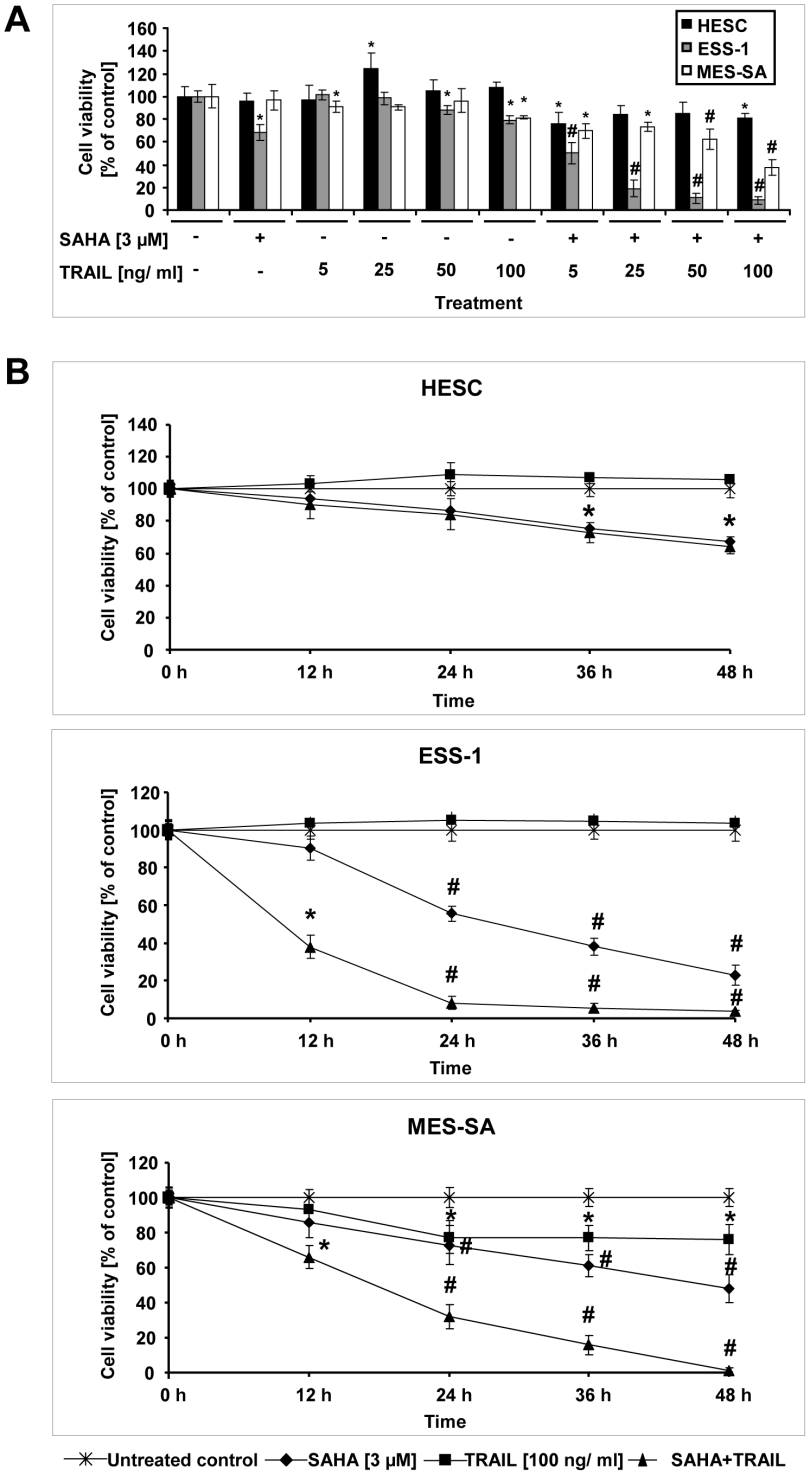
Cytotoxic effects of combined SAHA and TRAIL treatment in the cell lines ESS-1 and MES-SA. Cell viability (MTS) assays were performed after treatment of ESS-1 and MES-SA endometrial stroma sarcoma cells as well as HESC control cells for 24 hours with 3 μM of the HDAC inhibitor SAHA and/or different concentrations of human recombinant TRAIL (5, 25, 50, and 100 ng/ml) to determine the most effective cytotoxic concentration (A). Cell viability of ESS-1, MES-SA, and HESC control cells was determined in a time course experiment after treatment of cells for 0, 12, 24, 36, or 48 hours with 3 μM SAHA and/or 100 ng/ml TRAIL (B). The results in (A) and (B) are expressed as percentage of relative viability as compared to the untreated control. Cells were seeded at a density of 5×10^3^ cells per well. Each value represents the average of 3 independent experiments with 5 replicates each. Asterisks (* *p*<0.05) or number signs (# *p*<0.001) indicate statistically significant differences as compared to the untreated control.

In a first experiment, the most effective human recombinant TRAIL concentration for growth inhibition of ESS-1 and MES-SA uterine sarcoma cells as well as HESC cells was determined in a MTS assay. Five different TRAIL concentrations (5, 25, 50, and 100 ng/ml) were tested in the presence of 3 μM SAHA after 24 hours of treatment. As shown in Fig. 1A, even a TRAIL concentration as low as 5 ng/ml caused significant growth inhibition in comparison to untreated, or SAHA treated control cells, indicating a strong complementary interaction between the two compounds. These effects largely increased with higher TRAIL concentrations and were more effective for ESS-1 than for MES-SA cells after 24 hours. 3 μM SAHA and 100 ng/ml TRAIL were established as a final concentration for all further experiments performed as cell viability was reduced to about 9% (vs. ∼ 68% with single SAHA treatment) for ESS-1 cells, or to about 38% (vs. ∼ 97% with single SAHA treatment) in MES-SA cells, respectively. In addition, this concentration was assessed to be clearly synergistic for both cell lines (Fig. S1). Single TRAIL treatment in comparison was ineffective in reducing cell viability in tumor cells. Only the 100 ng/ml dose of TRAIL started to exert cytotoxic effects but overall these were modest (to ∼ 80% in both tumor cell lines vs. ∼ 108% in the HESC control cells).

For a further characterization of the cytotoxic action, a time course with both sarcoma cell lines and HESC control cells was performed over four time intervals (12, 24, 36, and 48 hours) (Fig. 1B). While only modest reduction in cell viability of HESC cells was observed even after 48 hours with any treatment (to ∼ 64% for SAHA or to ∼ 67% for SAHA/TRAIL treatment), the established working concentration of 3 μM SAHA and 100 ng/ml TRAIL proved to be very fast acting and effective on both investigated tumor cell lines with essentially all cells being eliminated. In comparison, SAHA treatment alone reduced cell viability to about 23 % in ESS-1 cells or to about 48% in MES-SA cells after 48 hours. Overall, both tumor cell lines demonstrated high sensitivity towards the combined treatment but ESS-1 cells responded more quickly with a more pronounced increase in cell death after already 24 hours.

### SAHA sensitizes uterine sarcoma cells to TRAIL-mediated apoptosis

**Figure 2:**
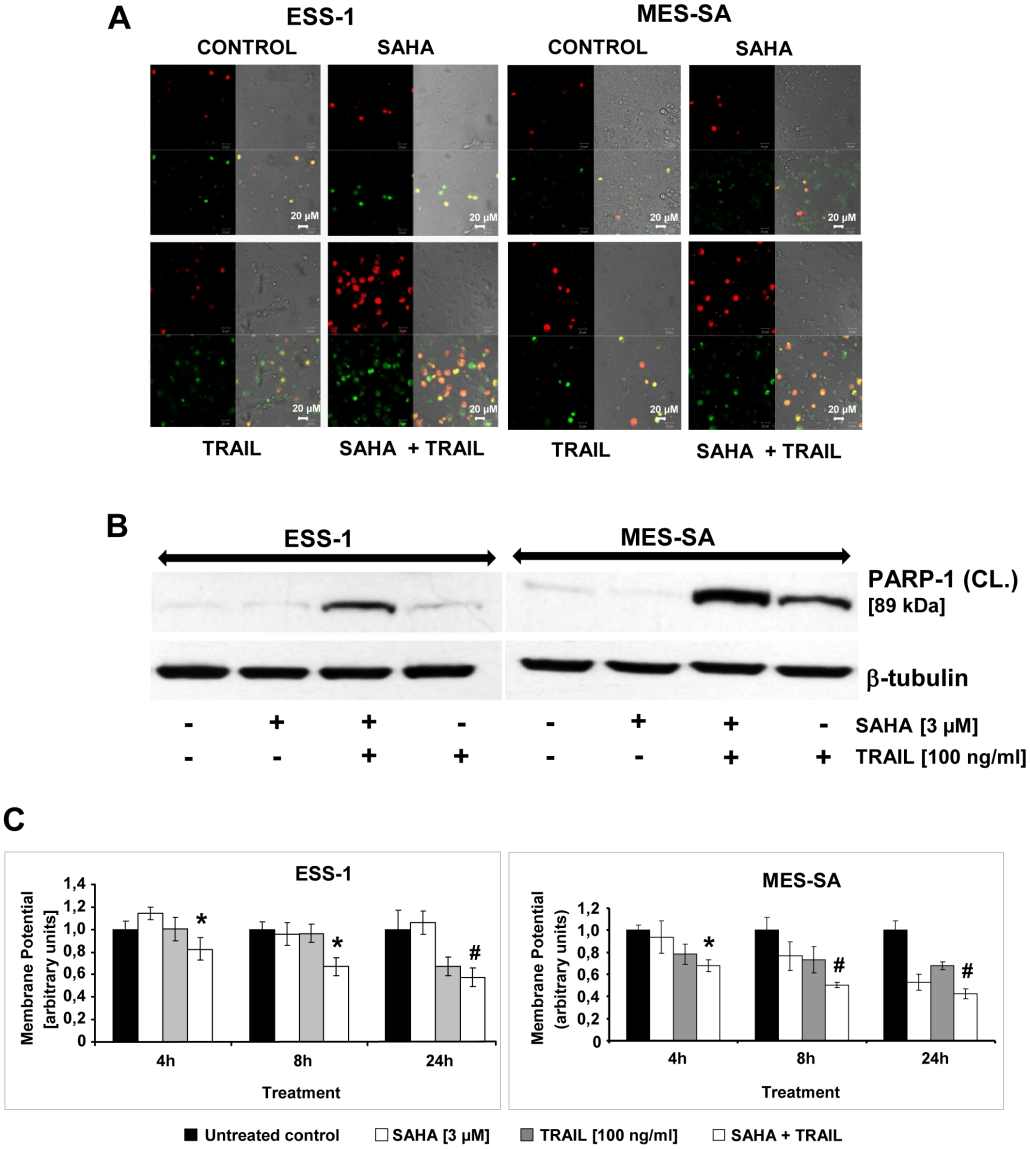
SAHA/TRAIL treatment induces apoptosis in uterine sarcoma cells involving the mitochondrial pathway. Confocal laser scanning microscopy of ESS-1 and MES-SA cells which were stained after 8 hours of 3 μM SAHA and/or 100 ng/ml TRAIL treatment with YoPro-1/PI in order to detect apoptotic and non-apoptotic cells (A). Control cells received neither SAHA nor TRAIL treatment. Red staining (PI) represents dead or necrotic cells, green staining (YoPro-1) represents apoptotic staining, merged (yellow/orange) staining represents secondary apoptotic cells (uptake of both dyes), and no staining represents living cells. Representative images of three independent experiments that were acquired at 505 to 530 nm for the green channel and 543 nm for the red channel are shown (magnification 40 x). (B) Western blot analysis of ESS-1 and MES-SA cells treated for 8 hours with 3 μM SAHA and/or 100 ng/ml TRAIL for PARP-1 in order to demonstrate apoptotis. Untreated cells were used as control. Cell extracts were prepared, subjected to SDS-PAGE (30 μg of protein; 4-12% Bis-Tris gel), and immunoblotted with antibodies against cleaved PARP-1 (89 kDa) and β-tubulin (for loading control). The presented 89 kDa PARP-1 fragment is only processed during induction of apoptosis but not necrosis [41]. (C) The mitochondrial membrane potential (Δψ_m_) was determined in uterine sarcoma cells (1×10^4^ cells per well) by JC-1 staining for confirming involvement of the intrinsic pathway of SAHA/TRAIL-induced apoptosis. Upon collapse of the Δψ_m_, JC-1 molecules can enter mitochondria where they form red J-aggregates. The red (∼590 nm; high Δψ_m_) to green (∼529 nm; low Δψ_m_) ratio therefore indicates the amount of apoptosis in SAHA/TRAIL-treated cells after 4, 8, and 24 hours in arbitrary units. Mitochondrial depolarization in dead cells or cells undergoing apoptosis is indicated by a decrease in the red/green fluorescence intensity ratio. Asterisks (* *p*<0.05) or number signs (# *p*<0.001) indicate statistically significant differences between the combined SAHA/TRAIL treatment and the untreated control.

YoPro-1/PI staining was used to assess the mode of cell death of synergistic SAHA (3 μM)/TRAIL (100 ng/ml) treatment (Fig. 2A). As depicted, ESS-1 and MES-SA cell populations exhibited a higher number of apoptotic and dead cells after only 8 hours of combined treatment in comparison to single SAHA or TRAIL treatment. Nevertheless, the percentage of double stained cells was more pronounced for ESS-1 than MES-SA cells at the analyzed time-point.

To confirm the induction of apoptosis in uterine sarcoma cells by combined SAHA/TRAIL treatment with a more specific marker, cleavage of PARP-1 was demonstrated by immunoblotting. As shown in Fig. 2B, a prominent 89 kDa fragment representing cleaved PARP-1, indicative for apoptotic cell death [41], appeared exclusively in cells treated for 8 hours with SAHA and TRAIL or single TRAIL treatment; single SAHA or no treatment provoked only a very slight band for cleaved PARP-1.

### SAHA and TRAIL treatment induces mitochondrial regulation of apoptosis in uterine sarcoma cells

Depolarization of Δψ_m_ has been correlated with the induction of cellular apoptosis. [21]. The membrane-permeant JC-1 dye is widely used in apoptosis studies to monitor mitochondrial membrane permeability. The quantitative analysis of JC-1–stained uterine sarcoma cells revealed a significant decrease in the red to green ratio in SAHA/TRAIL-treated cells after 4, 8, and 24 hours when compared with control cells, which was slightly higher in MES-SA cells than in ESS-1 cells (Fig. 2C). Significant loss of Δψ_m_ upon single SAHA treatment was observed only in MES-SA cells at later time-points which is consistent with previous reports of prevailing induction of autophagy in ESS-1 cells [16] but did not reach the level of the combined treatment. A similar ratio that was decreasing continuosly over time was also observed in single TRAIL treated MES-SA cells, whereas in ESS-1 cells a significant drop occurred only after 24 hours.

In conclusion, the measured changes of Δψ_m_ are indicative for induction of apoptosis via the intrinsic apoptotic pathway in both analyzed uterine cell lines.

### Cytofluorometric quantification of SAHA and TRAIL-induced apoptosis in uterine sarcoma cells

Independently from the measurement of effector caspases, and PARP-1 cleavage, we quantified the induction of SAHA/TRAIL-mediated apoptosis by bivariate AnnV/PI cytofluorometric analysis (Table 1, Fig. S3 and Text S1). Upon 4 hours of combined SAHA/TRAIL treatment about 4% apoptotic (AnnV-positive) in combination with 22% dead (PI-positive) ESS-1 cells and about 24% apoptotic in combination with 11% dead MES-SA cells could be detected. Regardless of the combined treatment, only single TRAIL treatment led to a higher percentage of apoptosis induction (∼23%). Apoptosis and cell death in single SAHA-treated ESS-1, MES-SA or untreated control cells was at a basal range of about 3% to 9%. After 24 hours of combined treatment, the situation dramatically changed and almost all ESS-1 cells (∼94%) stained highly positive for PI indicating cell death. In contrast, about 52% of MES-SA cells indicated apoptosis and about 34% cells were found dead at this time point. In addition, only the fraction of single SAHA-treated ESS-1 cells that underwent cell death gained a significant increase at 24 hours (∼53%). Induction of apoptosis reached about 26% in both cell lines by single TRAIL treatment and about 23% in MES-SA cells by single SAHA treatment.

**Table 1:** Quantitative bivariate AnnV/PI cytofluorometric analysis of apoptosis in SAHA and TRAIL-induced uterine sarcoma cells.

*	Cell-Line	ESS-1				MES-SA			
Time	Treatment/Staining	^#^CO	SAHA	TRAIL	^‡^S + T	^#^CO	SAHA	TRAIL	^‡^S + T
**4 h**	**Apoptotic cells (%)**±SD	**6.72**±1.80	**3.47**±0.50	**5.54**±0.83	**4.29**±0.07	**8.81**±1.75	**6.40**±0.47	**23.38**±0.67	**23.74**±0.28
	**Dead cells (%)**±SD	**7.85**±1.23	**4.93**±0.18	**16.10**±0.40	**21.61**±0.68	**3.52**±0.09	**2.64**±0.13	**9.99**±1.00	**11.19**±0.99
**24 h**	**Apoptotic cells (%)**±SD	**2.83**±0.15	**4.59**±2.61	**26.03**±0.58	**2.68**±0.37	**8.95**±0.49	**22.82**±2.24	**25.75**±0.64	**51.51**± 2.09
	**Dead cells (%)**±SD	**3.46**±0.16	**53.47**±1.06	**13.17**±0.52	**94.14**±0.11	**4.38**±0.44	**15.12**±0.66	**12.85**±0.67	**33.86**±2.79

*Presented is the mean ± SD of three independent experiments. **^#^** Untreated Control; ^‡^ Combined S(AHA) [3 μM] and T(RAIL) [100 ng/ml] treatment.

Collectively, the results revealed a more dominant but slower induction of apoptosis in MES-SA cells as compared to ESS-1 cells by combined SAHA/TRAIL treatment and confirmed largely the results gained by effector caspase and cell viability measurements.

### Comparison of combined SAHA and TRAIL with single SAHA-induced caspase activity in uterine sarcoma cells

**Figure 3:**
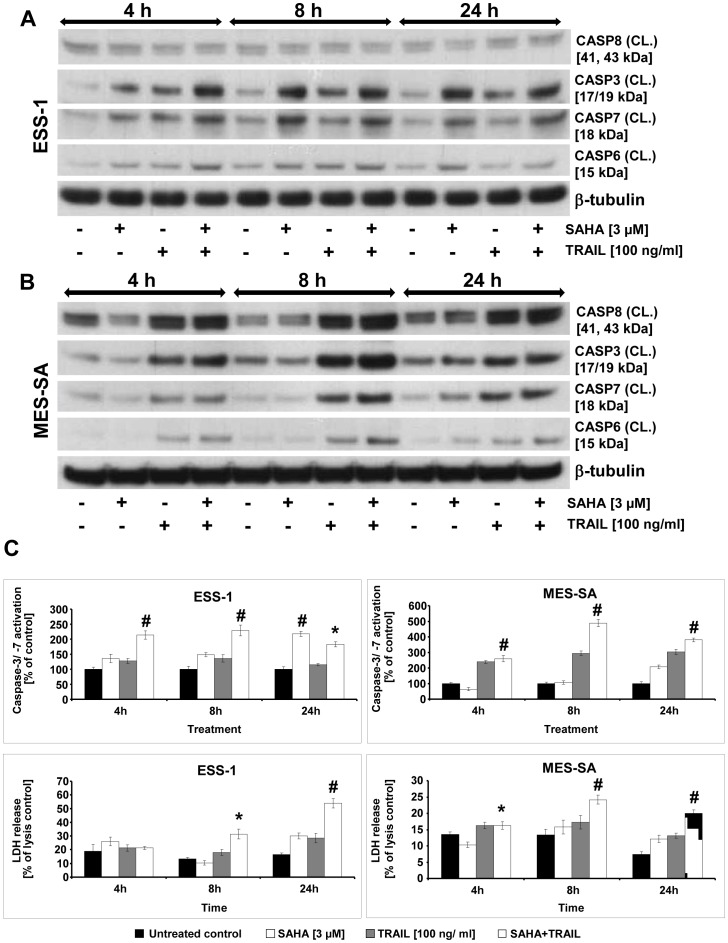
Time course of combined SAHA and TRAIL-induced caspase activation in uterine sarcoma cells. Western blot analysis of ESS-1 cells (A) and MES-SA cells (B) after treatment with 3 μM SAHA and/or 100 ng/ml TRAIL for 4, 8, and 24 hours to compare the induction of apoptosis. Untreated cells were used as control. Cell extracts were prepared, then 50 μg of protein were loaded onto a SDS-PAGE (12% Bis-tris gel) of each sample, and blotted onto nitrocellulose membrane. Subsequently, the membrane was incubated with the indicated antibodies against cleaved (CL.) caspases-3, -6, -7, -8, and β-tubulin (as loading control) followed by detection with a secondary hrp-coupled antibody. The molecular weights of presented bands are indicated in brackets. Note the weak expression of caspase-8 in ESS-1 cells (C) The amount of caspase-3 and -7 activation (Caspase-Glo 3/7 Assay; upper panel) and LDH release (CytoTox-ONE Homogeneous Membrane Integrity Assay; lower panel) of both uterine sarcoma cell lines was measured 4, 8, and 24 hours after treatment with 3 μM SAHA and/or 100 ng/ml TRAIL. The results are expressed as percentage of relative caspase-3/-7 activation or LDH release as compared to the untreated control or lysis control, respectively. Cells were seeded at a density of 5×10^3^ cells per well. Each value represents the average of 3 independent experiments with 5 replicates each. Asterisks (* *p*<0.05) or number signs (# *p*<0.001) indicate statistically significant differences between the combined SAHA/TRAIL treatment and the control.

In order to compare the effectiveness of the previously published single SAHA treatment [16] to apoptosis induction by SAHA/TRAIL or single TRAIL treatment, we monitored the activation of effector caspases-3, -6, and -7, as well as the the initiator caspase-8 in uterine sarcoma cells (Fig. 3). Time-points of 4, 8, and 24 hours after treatment were chosen for this analysis by using Western blot analysis (Fig. 3A and B) and the Caspase-Glo 3/7 assay in combination with LDH measurements (Fig. 3C) to assess the amount of cell-mediated cytotoxicity. Generally, at all analyzed time-points, we detected higher levels of cleaved executioner caspases in tumor cells that received the co-treatment of SAHA and TRAIL in both assays. In comparison, the activation was about two-fold higher in MES-SA cells than in ESS-1 cells. In both samples, peak activation of the analyzed executioner caspases was reached already after 8 hours of treatment (∼ 230% in ESS-1 and ∼ 490% in MES-SA cells compared to the untreated control in Fig. 3C). This caspase-3/-7 peak also coincided with a significant increase in LDH release in ESS-1 cells (∼ 31% of lysis control) and MES-SA cells (∼ 24% of lysis control) at this time-point, and considerably increased in ESS-1 cells up to 24 hours (∼ 54% of lysis control) whereas it declined in MES-SA cells. Single SAHA treatment only affected activation of caspase-3, -6, and -7 in an equal manner in both tumor cell lines (∼ 200% of untreated control in Fig. 3C) upon 24 hours of treatment. This delayed induction of apoptosis is consistent with the cell viability analysis shown in Fig. 1B and with previously published data [13]. Single TRAIL treatment (100 ng/ml) on the other hand led to a slight, non-significant but constant activation of the effector caspases above the control.

As expected from previous analyses, we found high levels of cleaved caspase-8 by Western blot analysis in co-treated or single TRAIL-treated MES-SA cells in comparison to untreated or single SAHA-treated cells at different time points (Fig. 3B). These were in consistency with the levels of executioner caspases with the exception of slightly higher caspase-8 activity in untreated MES-SA cells after 4 hours of treatment. Surprisingly, however, we only observed weak activation of caspase-8 in all samples derived from differentially treated ESS-1 cells (Fig. 3A).

### TRAIL resistance in uterine sarcoma cells is caused by reduced expression of apoptotic genes

**Figure 4:**
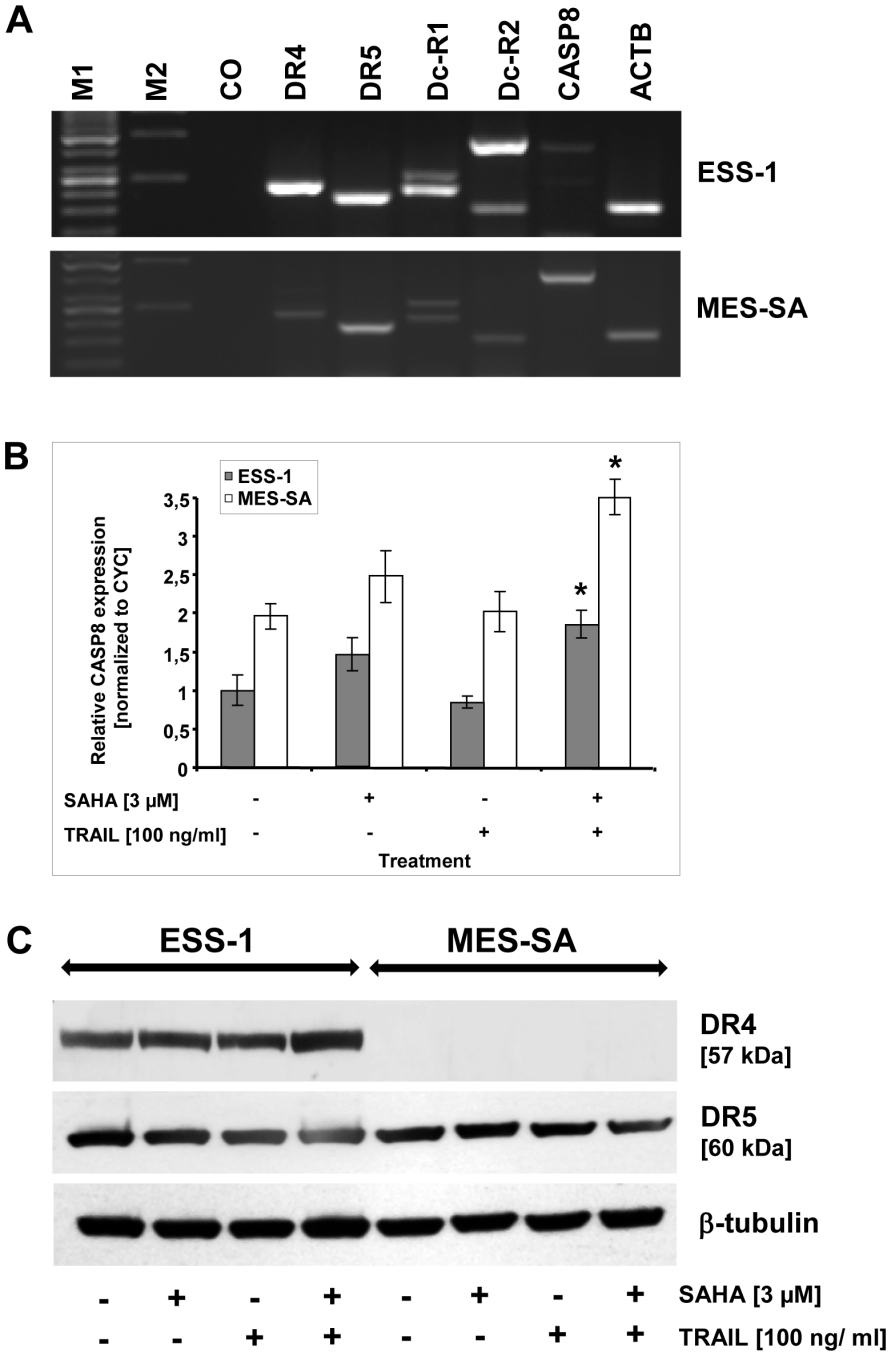
Reduced expression of caspase-8 in ESS-1 cells and DR4 (TRAIL-R1) in MES-SA cells. TRAIL receptors (TRAIL-R1/DR4 and TRAIL-R2/DR5), both TRAIL decoy receptors (Dc-R1, Dc-R2), and caspase-8 (CASP8) were amplified from cDNA in order to monitor defects in gene expression (A). Therefore, RNA of untreated cells was isolated, reversely transcribed, and subjected to qRT-PCR with primers binding to exonic sequences. PCR products were run on a 1.5% agarose gel, stained with ethidium bromide, and photographed. Amplification of beta-actin with or without genomic DNA served as a positive control (ACTB) or negative control (CO), respectively. Note the weaker bands for caspase-8 and DR4 in ESS-1 and MES-SA cells, respectively. The additional high molecular weight band for Dc-R2 in ESS-1 cells represents the genomic amplicon. M1, Gene ruler 50 bp DNA ladder; M2, λ*Bst*91I marker. (B) The relative caspase-8 expression of SAHA and/or TRAIL treated ESS-1, and MES-SA cells, was quantitated by qRT-PCR. Technical triplicate cycle threshold (ct) values of three biological replicates were normalized to cyclophillin (CYC) expression. Asterisks (* *p*<0.05) indicate statistically significant differences as compared to the untreated control. (C) Western blot analysis of ESS-1 and MES-SA cells treated for 8 hours with 3 μM SAHA and/or 100 ng/ml TRAIL. Untreated cells were used as control. Cell extracts were prepared, then 30 μg of protein subjected to SDS-PAGE (4 to 12% Bis-tris gel), and immunoblotted with the indicated antibodies against DR4 (TRAIL-R1), DR5 (TRAIL-R2) and β-tubulin as loading control. The molecular weights of presented bands are indicated in brackets.

In order to identify the cause of TRAIL resistance in the investigated uterine sarcoma cells, we investigated TRAIL receptor and caspase-8 expression at the mRNA and protein level (Fig. 4). First, both TRAIL receptors (DR4 and DR5), both TRAIL decoy receptors (Dc-R1, Dc-R2), and caspase-8 were amplified from cDNA of untreated cells. As displayed in Fig. 4A, only a slight band was detected for caspase-8 in ESS-1 cells and DR4 in MES-SA cells. No considerable difference was found for other transcripts.

The difference in caspase-8 transcript levels between ESS-1 and MES-SA cells were examined also by qRT-PCR in addition (Fig. 4B). In untreated MES-SA cells, expression levels of caspase-8 transcripts reached two times higher levels than those of ESS-1 cells which was still at least two times higher in case of the different treated cells. Upon treatment with SAHA alone or in combination with TRAIL, caspase-8 mRNA levels increased slightly in ESS-1 cells or significantly in MES-SA cells.

For confirmation of the results gained by qRT-PCR, we performed Western blotting for DR4 and 5 (Fig. 4C). As the results clearly confirm, no signal could be identified in MES-SA cells upon detection with a DR4 antibody in untreated or treated cells in contrast to a distinctive signal present in all analyzed ESS-1 samples. No detectable difference was found in both tumor cell lines for DR5 protein expression, however.

Overall, the results indicated decreased levels of expressed and cleaved caspase-8 in ESS-1 cells and significantly reduced or absent DR4 expression levels in MES-SA cells which correlated well in transcriptional and translational analyses.

### Hypermethylation of the promoter regions of apoptotic genes causes epigenetic silencing

**Figure 5:**
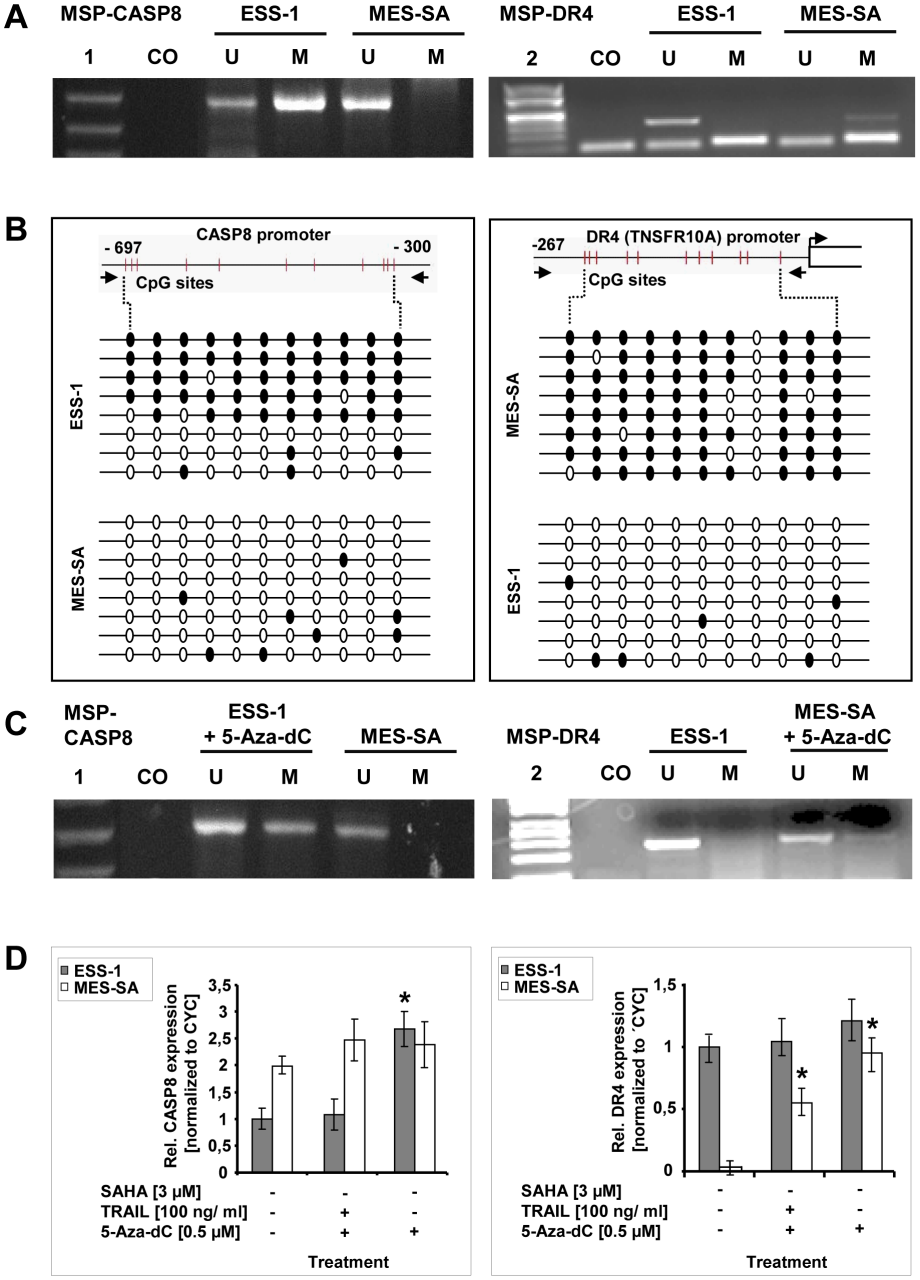
Promoter hypermethylation, and demethylation analysis of caspase-8 and DR4 genes in uterine sarcoma cells. Methylation-specific PCR (MSP) [36] of bisulfite treated genomic DNA, isolated from untreated ESS-1 and MES-SA cells, was performed of the promoter regions of *caspase-8* and *DR4* (*TNSFR10A*) for assessing the methylation status (A). Amplification with the help of primer pairs that either bind unmethylated (U) or methylated (M) DNA was conducted before the PCR product was visualized by gel electrophoresis. Unmodified genomic DNA was used as a negative control (CO). Note that the lower band in the MSP performed for *DR4* represents primers. Following DNA standards were used: (1) Gene ruler 50 bp DNA ladder; (2) λBst91I marker. (B) Bisulfite sequencing analysis [37], [38] of the *caspase-8* and *DR4* promoter regions in ESS-1 and MES-SA cells for identifying individual 5-methycytosine residues in genomic DNA. Eleven CpG sites located upstream of the transcription start site, between nucleotides – 300 and -697 for *caspase-8,* or nucleotides – 27 and – 267 for *DR4,* were analyzed for DNA methylation. Bisulfite converted genomic DNA, was amplified by PCR, subcloned, and sequenced by the Sanger method. The results of eight sequenced clones are depicted schematically for each promoter region and cell line, respectively. Methylated and unmethylated CpG nucleotides are presented as black or white circles, respectively. (C) MSP analysis as in (A) upon treatment of cells for 5 days with 0.5 μM 5-Aza-dC for examining induced alterations in genomic DNA methylation pattern for the CASP8 and DR4 promoter regions. Corresponding untreated cells were used as control. (D) Expression analysis of 5 day 5-Aza-dC treated ESS-1 and MES-SA cells for relative caspase-8 and DR4 expression by qRT-PCR. Triplicate ct values of three independent experiments were normalized to cyclophillin (CYC) expression and averaged. The depicted graphs represent the relative expression values as compared to the untreated control. Asterisks (* *p*<0.05) indicate statistically significant differences as compared to the untreated control.

We next analyzed, whether the expression of apoptosis inducing genes in the investigated uterine sarcoma cells was impaired by epigenetic silencing (Fig. 5). Previously, it has been shown that reduced caspase-8 expression in human cancer has been frequently caused by hypermethylation of the promoter region of the gene e.g. in Ewing tumor, neuroblastoma, malignant brain tumors, rhabdomyosarcoma or melanoma cells [42]. For a first analysis of the methylation status of *caspase-8* (*CASP8*) and *DR4* (*TNSFR10A*) genes in ESS-1 and MES-SA cells, respectively, we performed MSP using primers which corresponded to CpG-rich gene promoter regions [8], [36], [39] (Fig. 5A). Regarding the MSP for *caspase-8*, only a band for the unmethylated form could be amplified from bisulfite modified genomic DNA in MES-SA cells while a dominant band for the methylated, together with a weaker band for the unmethylated form, was observed in ESS-1 cells. The MSP for *DR4* demonstrated the exclusive presence of a band amplified by primers for unmethylated bisulfite-treated genomic DNA in ESS-1 cells and a weak band amplified by primers for methylated DNA in MES-SA cells.

In order to verify the results of MSP suggesting demethylation of CpG sites located in primer binding sites, bisulfite treated genomic DNA of the corresponding promoter regions was amplified, subcloned, and sequenced (Fig. 5B). Eleven CpG sites located upstream of the transcription start site, between nucleotides – 300 and – 697 for the *caspase-8* gene, or nucleotides – 27 and – 267 for the *DR4* gene, were analyzed. In ESS-1 cells, 5 out of 8 analyzed bisulfite treated sequences were found to be methylated for the caspase-8 promoter region while the remaining three sequences were found to be unmethylated; the CpG sites of control sequences derived from MES-SA cells showed no methylation in contrast. Bisulfite sequence analysis of the *DR4* promoter region confirmed the presence of predominantly methylated CpG sites in MES-SA cells which were encountered in a clearly unmethylated status in ESS-1 cells.

Upon treatment of cells for 5 days with 0.5 μM 5-Aza-dC, an inhibitor of DNA methyltransferase, we tested by using MSP whether the DNA methylation status of the promoter regions can be reversed (Fig. 5C). In both cases, we observed a more prominent PCR band amplified by the primers for the methylated form indicating a shift from the methylated towards the unmethylated form. Next, we examined if the mRNA expression of caspase-8 and DR4 could be restored by demethylating the target genes. We treated ESS-1 and MES-SA cells for 5 days with 0.5 μM 5-Aza-dC and performed qRT-PCR. Fig. 5D confirms that gene expression of caspase-8 in ESS-1 cells and DR4 in MES-SA cells can be restored with 5-Aza-dC treatment to a level comparable to the respective control cells. Interestingly, additional treatment of cells with TRAIL for 8 hours lowered the restored expression again to an intermediate level for DR4 in MES-SA cells or to the level of control cells for caspase-8 in ESS-1 cells. Collectively, MSP and bisulfite analysis in combination with qRT-PCR of 5-Aza-dC treated uterine sarcoma cells demonstrated silencing of gene expression pivotal for the induction of TRAIL-mediated apoptosis.

### Demethylation of apoptotic genes restores apoptosis in uterine sarcoma cells

**Figure 6:**
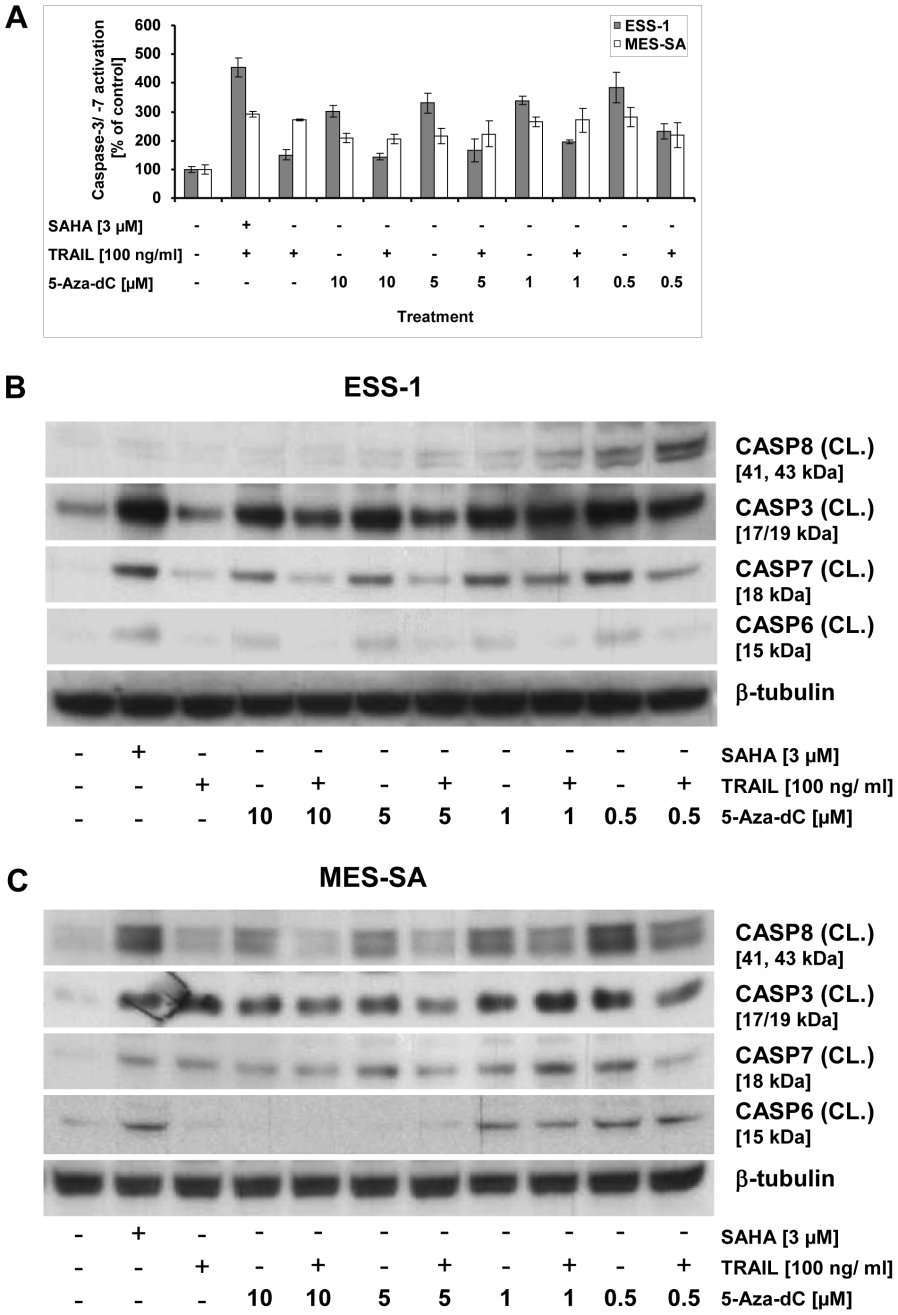
Reactivation of apoptosis through DNA demethylation by 5-Aza-dC in uterine sarcoma cells. Caspase-3 and -7 activation was measured in the uterine sarcoma cell lines, ESS-1 and MES-SA, upon DNA demethylation analysis (A). Therefore, treatment of cells with different concentrations (0.5, 1, 5, or 10 μM) of the DNA methylation inhibitor 5-Aza-dC was performed for 5 days with or without final addition of TRAIL (for 8 hours). Cells treated with 3 μM SAHA and/or 100 ng/ml TRAIL were included as positive controls. The cells received daily exchange of medium supplemented with fresh 5-Aza-dC whereas the control received no treatment at all. Western blot analysis of ESS-1 cells (B) and MES-SA cells (C) that were treated as in (A) for monitoring caspase activity upon DNA demethylation. Samples with 50 μg of protein were separated by SDS-PAGE (12% Bis-tris gel), transferred onto nitrocellulose membrane, and analyzed with antibodies against cleaved (CL.) caspases-3, -7, -6, -8, and β-tubulin as loading control. Untreated cells were used as control. The molecular weights of presented bands are indicated in brackets.

To further confirm our hypothesis that resistance of TRAIL-mediated apoptosis may be due to promoter methylation, we monitored activation of caspase-8 and executioner caspases (caspases-3, -6, and -7) in uterine sarcoma cells which were treated for 5 days with 5-Aza-dC (Fig. 6). Both, caspase-3/-7 activation assays (Fig. 6A) and Western blot analyses (Fig. 6B and C) were employed for this purpose. Both uterine cell lines were exposed to increasing concentrations of 5-Aza-dC from 0.5 to 10 μg/ml with or without additional treatment of 100 ng/ml TRAIL for 8 hours. Treatment with 0.5 μM of 5-Aza-dC turned out to be the most effective dose at which caspase-3/-7 induction climbed, in comparison to untreateted cells, to a 4-fold or 3-fold level in ESS-1 and MES-SA cells, respectively. These levels of activation were nearly equal to those induced by combined SAHA/TRAIL treatment. Surprisingly, the combination of TRAIL and 5-Aza-dC had a lesser apoptotic effect (∼ 50%) indicating that no external signal is required upon reactivation of epigenetically silenced gene expression. Immunoblotting confirmed the results gained by caspase-3/-7 induction for all executioner caspases in both analyzed tumor cell lines and for caspase-8 in MES-SA cells. In addition, in ESS-1 cells increasing re-induction of caspase-8 cleavage at lower 5-Aza-dC concentrations ranging from 1 to 0.5 μM was revealed (Fig. 6B).

The results above are consistent with caspase-8 and DR4 reexpression of 0.5 μM 5-Aza-dC treated cells gained by qRT-PCR (Fig. 5D). Therefore, we conclude that induction of apoptosis in the analyzed sarcoma cells is indeed hampered by transcriptional repression through DNA hypermethylation.

### Gene transfer of apoptotic genes restores apoptosis in uterine sarcoma cells

**Figure 7:**
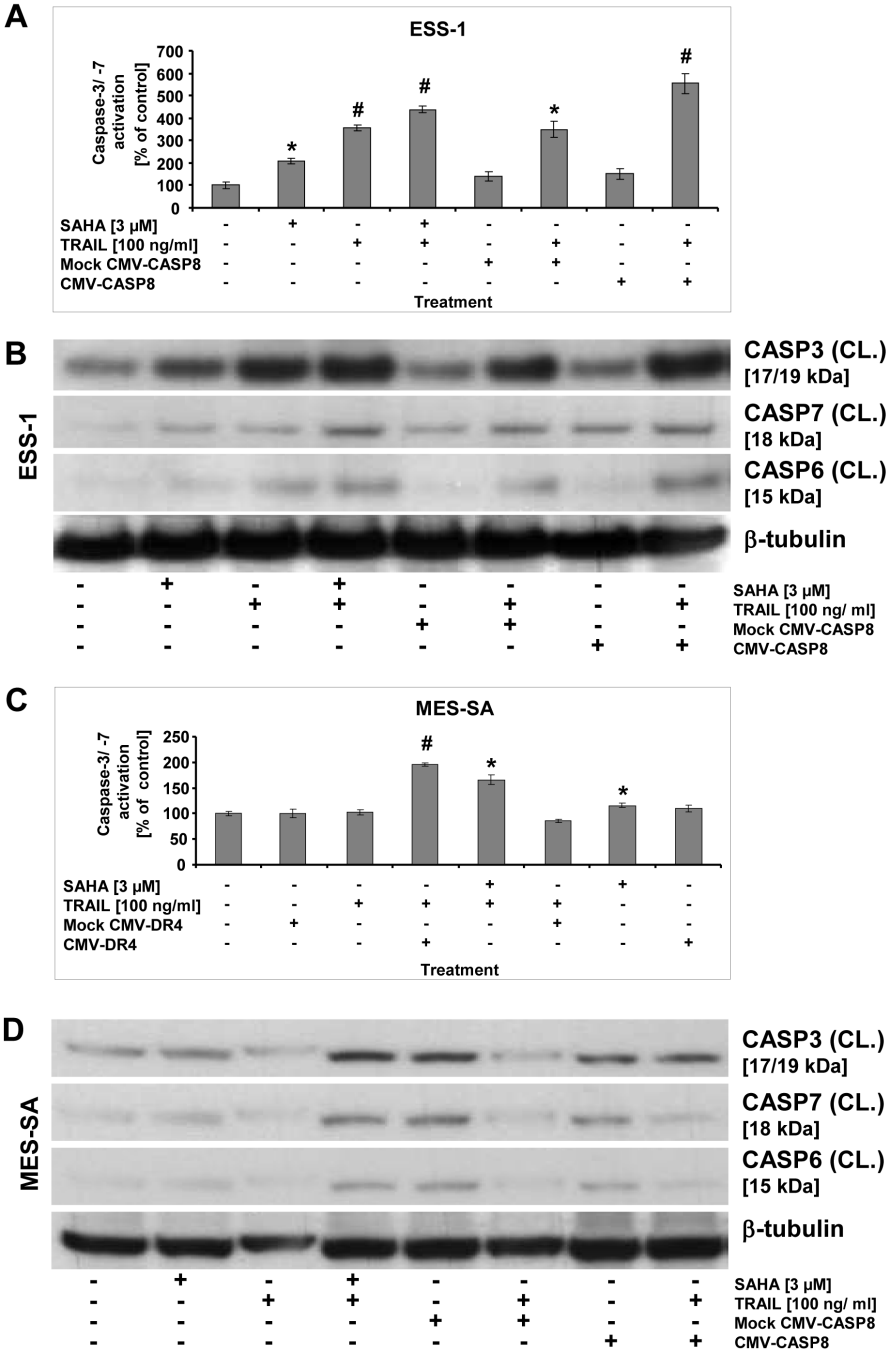
Reactivation of apoptosis by gene transfer in uterine sarcoma cells. Measurements of apoptosis in uterine sarcoma cells by caspase-3/-7 activation that was reinduced by gene transfer (A and C). ESS-1 (A) and MES-SA cells (C) were transfected with caspase-8 (A) or DR4 (C) expression plasmids driven by a CMV promoter, respectively, and were supplemented with or without TRAIL before caspase-3/-7 activation was measured 24 hours later. Controls were mock-transfected and treated with or without TRAIL. For comparison, cells that received 3 μM SAHA and/or TRAIL were measured. Presented is the relative caspase activation in percentage as compared to untreated control cells. Asterisks (* *p*<0.05) or number signs (# *p*<0.001) indicate statistically significant differences as compared to the untreated control. Western blot analyses of activated executioner caspases of ESS-1 cells (B) and MES-SA cells (D) in order to observe apoptosis reinduction upon gene rescue experiments as demonstrated in (A). Samples with 50 μg (B) or 30 μg (D) protein were immunoblotted and analyzed with antibodies against cleaved (CL.) caspases-3, -6, -7, and β-tubulin as loading control. Untreated cells were used as control. The molecular weights of presented bands are indicated in brackets.

Finally, we corroborated the crucial role of caspase-8 or DR4 in TRAIL-induced apoptosis by transient transfection of ESS-1 and MES-SA cells with full length cDNA expression constructs for caspase-8 or DR4, respectively (Fig. 7). Apoptosis detection of transfected cells was done again by observing induction of effector caspases through caspase-3/-7 activation assay (Fig 7A and C) as well as through immunoblotting of caspases-3, -6, and -7 (Fig.7 B and D) and by comparing it to those of mock-treated cells or cells that received SAHA and TRAIL. The analyses by both methods demonstrated that in both tumor cells lines, the transient restoration of the corresponding full length cDNAs in combination with single TRAIL treatment evoked reinduction of all analyzed effector caspases. The observed level of induction was even higher than that obtained by combined SAHA/TRAIL treatment (∼ 600% vs. 450% in ESS-1 and ∼175% vs. 150% in MES-SA cells). In contrast, no (additional) effect was gained by single mock transfection with or without TRAIL combination.

Together, these results prove induction of DNA hypermethylation as an essential alteration which prevents apoptosis in the tested uterine sarcoma cells which can be overcome by reexpression of silenced tumor suppressor genes through demethylation or gene transfer.

## Discussion

Our previous studies showed that SAHA and/or other HDAC inhibitors could be potential candidates for the treatment of ESS and uterine sarcomas in general. During further experiments exploring the molecular mechanisms of SAHA-induced apoptosis or autophagy, we noticed a remarkable enhanced cytotoxic effect on both uterine sarcoma cell lines when TNF-α was included in our experiments. In order to exploit this finding, we tested whether also the clinically more advantageous TRAIL elicits a similar response, as TNF-α and TRAIL share common signaling pathways in apoptosis [20]. Recently, due to a number of studies, TRAIL has gained a lot of attention, because of its apoptosis-inducing capability in many tumor cells without harming non-malignant cells significantly. These attributes have led to current clinical testing of TRAIL as a novel encouraging therapeutic agent against cancer [23]. Thus, an effective TRAIL therapy would be preferable over recombinant TNF-α treatment due to less side effects and the availability of agonistic antibodies with extended biological half-life [17]. In addition, in previous phase II clinical trials several evidences led to the conclusion that HDAC-based combination therapies would be more effective for the treatment of solid cancer than monotherapeutic HDAC inhibitor treatment [43].

Therefore, the aim of the current study was to evaluate the rationale of using SAHA and/or TRAIL therapy for the treatment of uterine sarcomas with an emphasis on ESS. The demonstrated experiments proved the efficient and complete elimination of both tumor cell lines within 24 to 48 hours by using the combination of SAHA with TRAIL. For this reason, all experiments had to be performed at early time-points in this study and might thus, besides the use of different techniques, explain several differences to data that were obtained in our previous studies. In fact, in comparison to single SAHA treatment, the apoptotic response was induced very fast and was higher in ESS-1 cells than in MES-SA cells. Untreated control cells or cells treated with TRAIL only, however, exhibited negligible effects of apoptosis. This was not unexpected, since resistance to cell death induction has been recognized as a hallmark of cancer. Very often, the knowledge about the underlying molecular events regulating different cell death mechanisms has opened new possibilities for targeted interference with these pathways. Therefore, we decided to analyze the cause of TRAIL resistance in both investigated sarcoma cell lines and thereby gain insight into the pathogenetic molecular mechanisms.

Several causes of genetic origin resulting in resistance to apoptosis induced by death receptors have been described in different types of tumors. Among those, mutations in death receptor and caspase-8 genes, overexpression of inhibitory molecules such as soluble death receptors, decoy receptors, and FLIP have been identified. In addition, epigenetic silencing of members of the death receptor pathway via DNA methylation can also occur. In cancer cells, methylation of CpG islands of tumor suppressor genes occurs more frequently and correlates with transcriptional repression. Especially, inactivation of the caspase-8, Fas and DR4/TRAIL-R1 genes either by DNA methylation or mutation as part of the malignant process have been detected in cancer cells, and are thought to contribute to carcinogenesis [44].

In the present study, uterine sarcoma cells were found to be highly resistant to death receptor-induced apoptosis by single TRAIL treatment. The mechanisms responsible for this resistance were found to include silencing of caspase-8 in ESS-1 cells and TRAIL-R1 genes in MES-SA cells, by DNA hypermethylation of their promoter regions. As a consequence, we observed reduced caspase-8 or DR4 expression which clearly explains how TRAIL generated signals are not at all, or less efficiently transduced, and how these tumor cells escape death receptor-induced apoptosis. Nevertheless, we cannot exclude any additional defects in the activation of pro-caspase-8 which might also influence TRAIL signalling in the case of ESS-1 cells. DR5 expression, however, and the activation of caspase-6 which is directly implicated in this process [45], were not altered in any tumor cell line. The crucial role of both tumor suppressor genes was further supported by the restoration of TRAIL-sensitized apoptosis upon recombinant overexpression of caspase-8 and DR4 or re-induced expression by DNA demethylation. Silencing of tumor suppressor genes is often an early event in the development of human cancer. Therefore, if these findings can also be confirmed for the *in vivo* situation, the detection of sites of altered DNA methylation would constitute promising molecular markers. They could be useful in early cancer detection and in monitoring disease progression as well as responses to treatment. Silenced expression of the DR4 gene has been previously reported for other tumor cells e.g. in ovarian tumors and cell lines [46], melanoma cell lines [33], and astrocytic gliomas [47]. Hypermethylation of the caspase-8 gene has been detected in childhood neuroblastomas [37], lung tumors and cell lines [48], pedriatric tumors and cell lines [49], breast cancer cells [50], and several others.

Previous reports have shown that the simultaneous administration of SAHA and TRAIL significantly increased the expression of the TRAIL death receptors DR4/DR5 and mitochondrial damage of TRAIL in several cell lines [51], [52]. Consequently, the pre-treatment or co-treatment with SAHA or other HDAC inhibitors could augment the cytotoxic and apoptotic effect of TRAIL. In this study, we did not confirm any significant change in TRAIL receptor expression effectuated by SAHA/TRAIL co-treatment in MES-SA cells. In ESS-1 cells the slight upregulation of DR4 expression, as seen in Fig. 4B, was consistent with previous reports. Nonetheless, in the case of MES-SA cells it seems that negligible SAHA-induced upregulation of DR4 expression which is undetectable by immunoblotting (Fig. 4C) led to the faint increase in caspase-8 expression as observed by qRT-PCR (Fig. 4B). Overall this difference in DR4 expression might also explain the delayed induction of cell death in MES-SA cells as compared to ESS-1 cells.

To investigate whether the intrinsic pathway of apoptosis was employed to augment the caspase cascade via the mitochondrium, we measured the Δψ_m_ in the sarcoma cells. Indeed, we observed a significantly increased drop in Δψ_m_, which is in agreement with other previous reports that included TRAIL-induced apoptosis [28], [29], [51], [52]. The important role of mitochondria-mediated apoptotis induction was furthermore confirmed by cytofluorometric analysis in our experiments. Therefore, we examined next whether the higher dissipated membrane potential led also to a higher engagement of the downstream effector caspases -3, -6, -7, and -9 (Fig. S2). In both tumor cell lines, pretreatment with the pan-caspase inhibitor, or with preferential caspase-inhibitors for caspases -9, -8, -3 and -7, reduced the cytotoxicity of SAHA and TRAIL, indicating the involvement of these caspases. Nevertheless, the conclusively low apoptotic potential of caspase-3 and -7 compared to the high potential of caspase-9 in ESS-1 cells, supports the previously revealed cytotoxic role for autophagy in those cells [16]. As the activation of caspase-9 by cytochrome-C release from the mitochondrium does not seem to lead to consequently higher caspase-3 and -7 engagement, one could assume that caspase-9 was also involved in induction of autophagy via a cross-talk mechanism. Alternatively, the discrepancy between caspase-3 and -7 independent SAHA/TRAIL cytotoxic effects and the relatively high observed levels of apoptosis could also be explained by mediation through the release of the mitochondrial flavoprotein, AIF (Apoptosis inducing factor) [53]. During apoptotic signaling, AIF is released from the mitochondria by permeabilization of the mitochondrial membrane, and translocates to the nucleus. In the nucleus, this effector molecule was found to induce programmed cell death by triggering chromatin condensation and DNA fragmentation in a caspase-independent mode. Cell-type specific caspase-independent cytotoxic effects of SAHA have been reported previously in this context [54].

Collectively, these *in vitro* results suggest that a combination of HDAC inhibitor and TRAIL-stimulatory agents may be therapeutically advantageous. Further experiments for clarification of the cytotoxicity improving molecular mechanisms in MES-SA cells might add to our understanding. It remains to be proven whether the combination of SAHA (Vorinostat) with TRAIL receptor agonists will prove effective in xenograft models as tested for SAHA treatment previously [13], before clinical trials can be targeted. Opposed to the use of purified recombinant ligands of the TRAIL receptor as TRAIL or TNFα, a combination of Vorinostat and an agonistic monoclonal anti-TRAIL antibody (e.g. DR4/TRAIL-R1: Mapatumumab or DR5/TRAIL-R2: Lexatumumab, MD5-1) could also be tested, alternatively [55]. This synergistic combination accomplished at least in mice a favourable reduction of established mammary tumors in the absence of significant toxicity, and provided the advantage of increased effect by extended biological stability in the organism [56].

Another, yet unexplored possibility, presents the possibility of investigating therapeutic effects of the DNA methyltransferase inhibitor 5-Aza-dC (decitabine) alone or in combination with HDAC inhibitors [57]. Initial experiments in our study, where silencing of gene expression of caspase-8 or DR4 was reversed (Fig. 5D) and apoptosis induction (Fig. 6A), even without TRAIL addition, was restored by 5-Aza-dC treatment, provided such evidence. In this context, further experiments exploring whether altered expression levels of DNA methyltransferases cause aberrant DNA hypermethylation in uterine sarcomas will be necessary.

## Conclusions

In summary, we provide here *in vitro* molecular evidence that epigenetic silencing of the uterine sarcoma cell lines, ESS-1 and MES-SA, is not only caused by upregulation of HDACs but also by hypermethylation of promoter regions of tumor suppressor genes. Consequent resistance can be overcome by HDAC inhibitor (SAHA) treatment which resensitizes the tumor cells for TRAIL-mediated apoptosis signaling. These findings could provide the basis for further preclinical evaluation of patients with uterine sarcoma by HDAC inhibitors in single or combined therapy.

## Supporting Information

Figure S1 **Assesment of synergistic effects of SAHA and TRAIL treatment on uterine sarcoma cell lines.** Synergistic, additive, and subadditive effects of combined SAHA [3 μM] and TRAIL treatment [different doses from 5 to 100 ng/ml] on the cell viability of the uterine sarcoma cell lines ESS-1 and MES-SA represented by the O/E ratio [O/E<0.8, synergistic; O/E  =  0.8–1.2, additive; O/E>1.2 subadditive]. The ratio was calculated using an additive model [40].
(TIF)

Figure S2 **Determination of caspase dependency of SAHA and TRAIL-induced apoptosis and cytotoxicity.** Assay for caspase-3 and -7 activation (A) (Caspase-Glo 3/7 Assay; upper panel) and cell viability (MTS assay; lower panel) of the uterine sarcoma cell lines ESS-1 and MES-SA (B) in the the presence of 10 μM caspase inhibitors. Inhibitors were added to cells 1 hour before the 24 hour SAHA/TRAIL treatment was initiated. Z-VAD-FMK, caspase-family inhibitor; Z-DEVD-FMK, caspase-3 and -7 inhibitor; Z-IETD-FMK, caspase-8 inhibitor; Z-LEHD-FMK, caspase-9 inhibitor.
(TIF)

Figure S3 **Quantitative bivariate AnnV/PI cytofluorometric analysis of apoptosis in SAHA and TRAIL-induced uterine sarcoma cells.** Apoptosis induced by 3 μM SAHA and/or 100 ng/ml TRAIL was quantified by staining cells after 4 and 24 hours of treatment with AnnV and PI (A) followed by cytofluorometric bivariate analysis (see also Table 1). Intact cells (PI negative, AnnV-FITC negative; lower left quadrant), early apoptotic cells (PI negative, AnnV-FITC positive; lower right quadrant), and late apoptotic cells (PI positive, AnnV-FITC positive; upper right quadrant), as well as necrotic or dead cells (PI positive, AnnV-FITC negative; upper left quadrant) can be differentiated.
(TIF)

Text S1 **Quantitative bivariate AnnV/PI cytofluorometric analysis of apoptosis in SAHA and TRAIL-induced uterine sarcoma cells.**
(DOC)
