# Supplementary figures and images for: Epigenetic Silencing of Apoptosis-Inducing Gene Expression Can Be Efficiently Overcome by Combined SAHA and TRAIL Treatment in Uterine Sarcoma Cells
Source: PLoS One. 2014 Mar 11;9(3):e91558. doi: 10.1371/journal.pone.0091558 (PMC3950220; doi:10.1371/journal.pone.0091558)

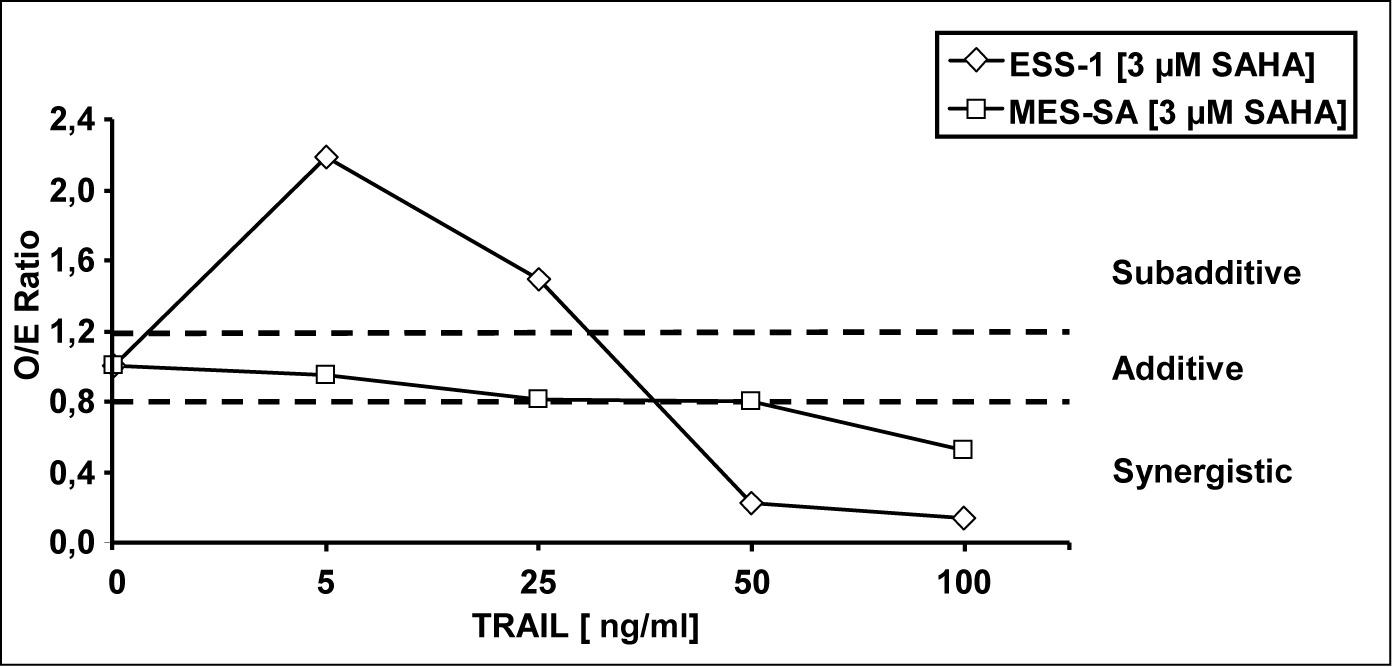

Supplement: Figure S1 — Assesment of synergistic effects of SAHA and TRAIL treatment on uterine sarcoma cell lines. Synergistic, additive, and subadditive effects of combined SAHA [3 μM] and TRAIL treatment [different doses from 5 to 100 ng/ml] on the cell viability of the uterine sarcoma cell lines ESS-1 and MES-SA represented by the O/E ratio [O/E<0.8, synergistic; O/E = 0.8–1.2, additive; O/E>1.2 subadditive]. The ratio was calculated using an additive model [40]. (TIF) [file pone.0091558.s001.tif]

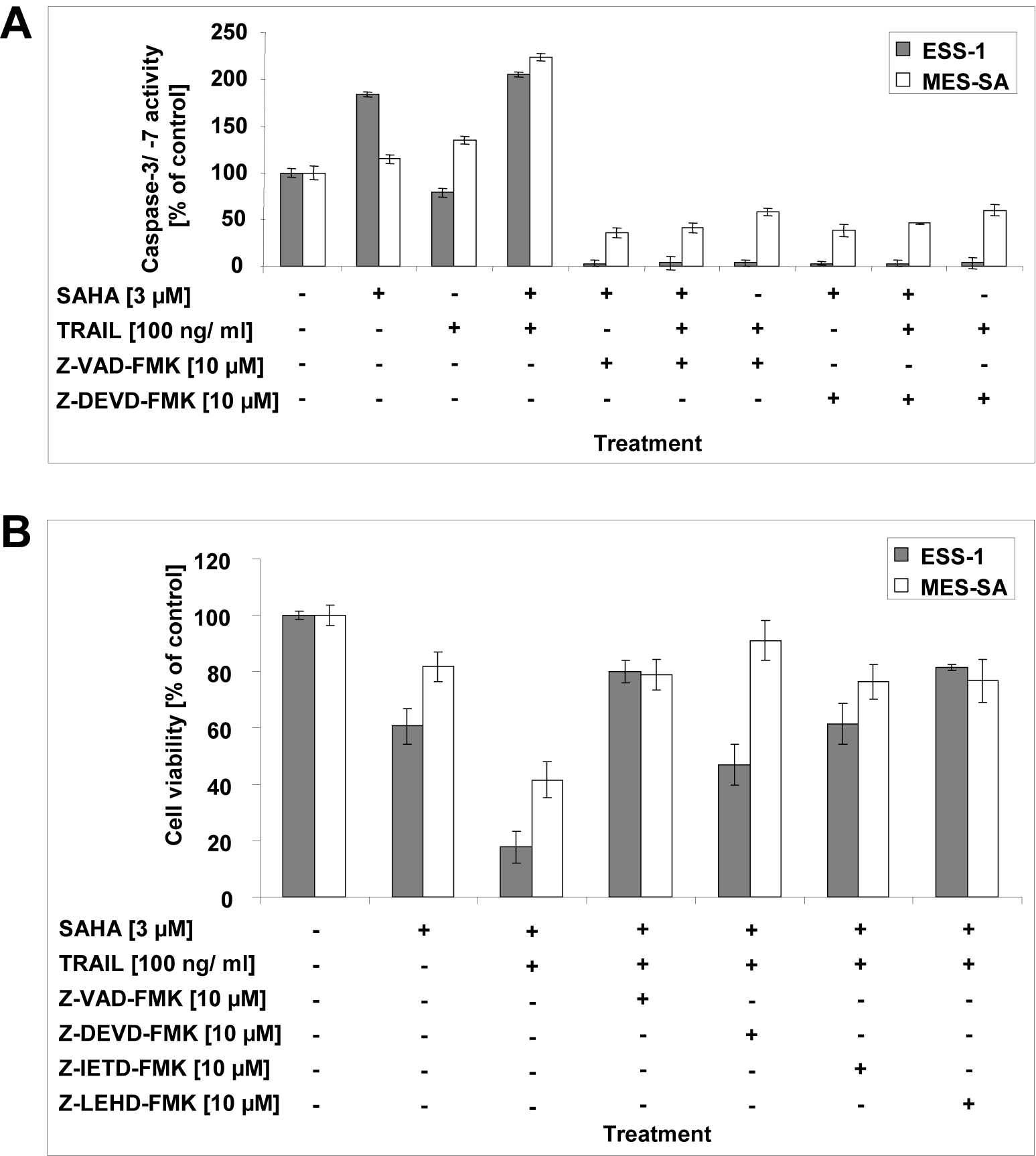

Supplement: Figure S2 — Determination of caspase dependency of SAHA and TRAIL-induced apoptosis and cytotoxicity. Assay for caspase-3 and -7 activation (A) (Caspase-Glo 3/7 Assay; upper panel) and cell viability (MTS assay; lower panel) of the uterine sarcoma cell lines ESS-1 and MES-SA (B) in the the presence of 10 μM caspase inhibitors. Inhibitors were added to cells 1 hour before the 24 hour SAHA/TRAIL treatment was initiated. Z-VAD-FMK, caspase-family inhibitor; Z-DEVD-FMK, caspase-3 and -7 inhibitor; Z-IETD-FMK, caspase-8 inhibitor; Z-LEHD-FMK, caspase-9 inhibitor. (TIF) [file pone.0091558.s002.tif]

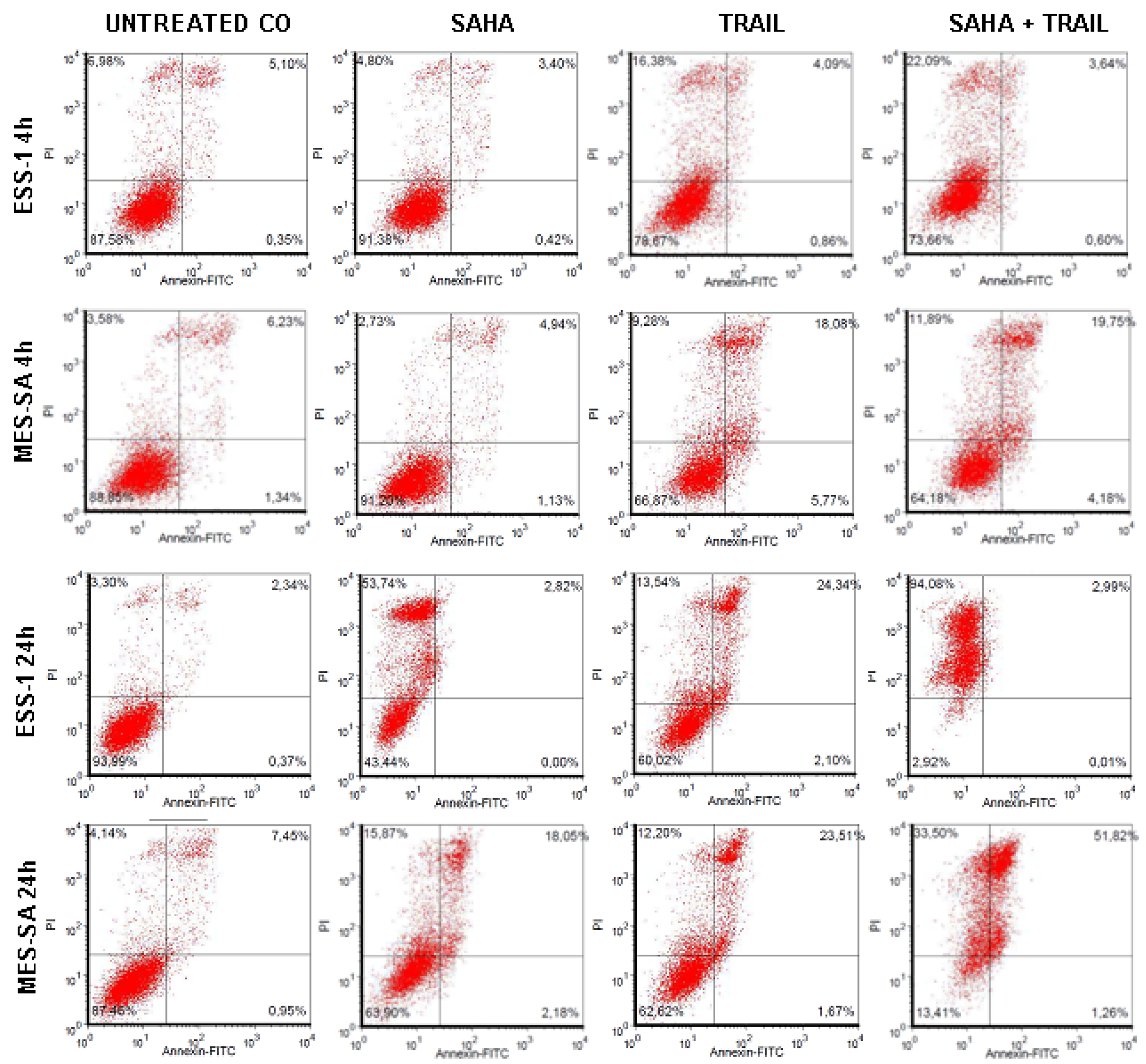

Supplement: Figure S3 — Quantitative bivariate AnnV/PI cytofluorometric analysis of apoptosis in SAHA and TRAIL-induced uterine sarcoma cells. Apoptosis induced by 3 μM SAHA and/or 100 ng/ml TRAIL was quantified by staining cells after 4 and 24 hours of treatment with AnnV and PI (A) followed by cytofluorometric bivariate analysis (see also Table 1). Intact cells (PI negative, AnnV-FITC negative; lower left quadrant), early apoptotic cells (PI negative, AnnV-FITC positive; lower right quadrant), and late apoptotic cells (PI positive, AnnV-FITC positive; upper right quadrant), as well as necrotic or dead cells (PI positive, AnnV-FITC negative; upper left quadrant) can be differentiated. (TIF) [file pone.0091558.s003.tif]
