# Supplementary material for: Epigenetic Silencing of Apoptosis-Inducing Gene Expression Can Be Efficiently Overcome by Combined SAHA and TRAIL Treatment in Uterine Sarcoma Cells
Source: PLoS One. 2014 Mar 11;9(3):e91558. doi: 10.1371/journal.pone.0091558 (PMC3950220; doi:10.1371/journal.pone.0091558)
Supplement: Text S1 — Quantitative bivariate AnnV/PI cytofluorometric analysis of apoptosis in SAHA and TRAIL-induced uterine sarcoma cells. (DOC) [file pone.0091558.s004.doc]

**Text S1.** **The effect of caspase inhibitors on SAHA and TRAIL-induced apoptosis.** To study the pathways involved in SAHA/ TRAIL-induced apoptosis, we repeated the Caspase-Glo 3/ 7 and cell viability assays in the presence of several caspase inhibitors in both tumor cell lines (Fig. S2A and B). Caspases-8, -9, -3 and -7 were analyzed since they are situated at pivotal junctions in apoptosis pathways. After 24 hours of treatment, activation of caspases-3 and -7 was similarly completely abrogated by co-treatment with the broad spectrum caspase inhibitor (Z-VAD-FMK) and the preferential caspase-3 and -7 inhibitor (Z-DEVD-FMK) in ESS-1 cells; In MES-SA cells this activation was decreased from about 200% to about 50% of the control (Fig. S2A). This clearly indicated that all measured apoptotic activity was transmitted via caspases-3 and-7 in both cell lines and suggested that there is residual, caspase-independent apoptosis induction in MES-SA but not in ESS-1 cells.

The cytotoxic effect exerted by the SAHA/ TRAIL combination could be prevented by the broad spectrum caspase inhibitor and the preferential caspase-9 inhibitor (Z-LEHD-FMK) in ESS-1 cells (Fig. S2B). A modest protective effect was instead observed in the presence of a preferential inhibitor of caspase-8 (Z-IETD-FMK) activity but no effect in the presence of the DEVD-dependent caspase inhibitor. With the exception of a modest blocking action of the preferential caspase-3 and -7 inhibitor, no increased viability of any applied inhibitor could be observed for MES-SA cells. The finding in ESS-1 cells suggests that activation of caspase-9 is important in order to enhance cell death upon SAHA/ TRAIL treatment. Interestingly, inhibition of caspases-8, -3 and -7 seemed to have a lesser cytoprotective effect which supports a role for caspase-independent cytotoxic mechanisms like autophagy as previously reported [16].
